# Sinful Foods: Measuring Implicit Associations Between Food Categories and Moral Attributes in Anorexic, Orthorexic, and Healthy Subjects

**DOI:** 10.3389/fnut.2022.884003

**Published:** 2022-06-13

**Authors:** Clara Lakritz, Lola Tournayre, Marilou Ouellet, Sylvain Iceta, Philibert Duriez, Vincent Masetti, Jérémie Lafraire

**Affiliations:** ^1^Centre de Recherche de l’Institut Paul Bocuse, Lyon, France; ^2^Laboratoire Parcours Santé Systémique, Université Claude Bernard Lyon 1, Lyon, France; ^3^GR2TCA-Loricorps, Groupe de Recherche Transdisciplinaire des Troubles du Comportement Alimentaire, Université du Québec à Trois-Rivières, Trois-Rivières, QC, Canada; ^4^Centre de Recherche de l’Institut Universitaire en Santé Mentale de Montréal (CR-IUSMM—CIUSSS Est de Montréal), Montreal, QC, Canada; ^5^Centre Référent pour les TCA, Centre Intégré de l’Obésité, Hospices Civils de Lyon, Lyon, France; ^6^Centre de Recherche de l’Institut Universitaire de Cardiologie et de Pneumologie de Québec-Université Laval, Quebec City, QC, Canada; ^7^GHU Paris Psychiatry and Neurosciences, Clinic of Mental Illnesses and Brain Disorders, Paris, France; ^8^INSERM U1266, Institute of Psychiatry and Neuroscience of Paris (IPNP), University of Paris, Paris, France; ^9^Clinea Psychiatrie France, Paris, France

**Keywords:** food categorization, moral judgment, cognition, eating disorders, anorexia nervosa, orthorexia nervosa

## Abstract

Recently, neurocognitive studies have shown that food categorization is sensitive to both the properties of the food stimuli (e.g., calorie content) and the individual characteristics of subjects (e.g., BMI, eating disorders) asked to categorize these stimuli. Furthermore, groups of patients with eating disorders (ED) were described as relying more on moral criteria to form food categories than were control subjects. The present studies built on these seminal articles and aimed to determine whether certain food properties might trigger moral categories preferentially in subjects suffering from ED and in the general population. Using a Go/No-Go Association Task, Study 1 focused on the extent to which food categories are laden with moral attributes in ED patients compared to control subjects. Study 2 was a follow-up with a different design (an Implicit Association Test), another food variable (calorie content), and two non-clinical subgroups (orthorexic and healthy control subjects). Results revealed for the first time implicit associations between food variables cueing for energy density and moral attributes in the general population, the population suffering from anorexia nervosa, and subjects suffering from disordered eating such as orthorexia nervosa. These findings suggest that moralization of food is a pervasive phenomenon that can be measured with methods reputed to be less vulnerable to self-presentation or social desirability biases.

## Introduction

Categorization is a fundamental ability that we rely on to organize sensory information into entities or categories of entities we might refer to. From such categories, we then generalize information to novel instances and act accordingly. For example, if an object is categorized as a blackberry, you are entitled to ascribe the edibility property to that object and then decide to eat it (1). Recent studies that investigated the nature of food categorization revealed that food categorization is far from simple and that the term actually uncovers manifold processes: from early and automatic discrimination of food depending on the sensory properties (2) to the building of elaborate morally-laden conceptual representations about foods (3). A further complication comes from the fact that food categorization seems very sensitive to both the properties of the food stimuli and the individual characteristics of subjects asked to categorize these stimuli.

At a very early stage of cognitive processing, the mere sight of food triggers a wide range of physiological, emotional, and cognitive reactions (4). For instance, in an electroencephalogram (EEG) study using visual evoked potentials, Toepel et al. (2) obtained evidence of early discrimination of subclasses of food images by manipulating their reward value (e.g., low fat food versus high fat food). They identified two discrimination stages: an early stage of categorization at ∼165 milliseconds (ms) and a second at ∼300 ms post-stimulus. The calorie content and the degree to which the food has been processed are also rapidly discriminated by the cognitive system. Analyzing event-related potentials, Pergola et al. (5) evidenced different neuronal activity depending on the degree of food processing and calorie content: natural (e.g., an apple) versus processed (e.g., lasagna).

In addition to the properties of the food, an individual’s characteristics influence food categorization as well. In the EEG study mentioned above, Pergola et al. (5) showed that the distinctive neuronal activity underpinning food processing is modulated by the body mass index (BMI) of participants. Specifically, they investigated the N400 amplitude and latency in response to food stimuli. N400 amplitude and latency reflect the incongruence or congruence between stimuli, and is measured by placing electrodes at specific locations on the scalp. Its amplitude and latency reflect the strength of the signal and the delay between the stimuli and the signal, respectively (6–8). In their study these stimuli were photographs depicting either a natural or a processed food (e.g., pineapple or pizza, respectively) and sentences that described either a sensory attribute (e.g., “It tastes sweet”) or a functional attribute defined as the context in which the food is eaten (e.g., “It is suitable for a wedding meal”). In the task, a sentence was followed by an image, and the sentence-image pairs were either congruent (“It tastes sweet” with pineapple) or incongruent (“It tastes salty” with pineapple). Results revealed modulations of N400 amplitude and latency caused by sensory-functional primes only for processed food (e.g., lasagna) in participants with obesity, whereas only for natural food in underweight participants (e.g., an apple).

Furthermore, interactions between these two types of variables that influence food categorization, namely those cueing energy density and an individual’s characteristics have been recently evidenced in behavioral studies. Coricelli et al. (9) conducted an exploratory analysis that revealed that restrained eaters (individuals who strictly control their tendency to eat for an extended period to lose or maintain body weight) were significantly slower at categorizing processed food as such compared to unrestrained eaters. The authors explained this effect by referring to work conducted by Papies et al. (10) who put forward that in restrained eaters, the attraction of food palatability might have interfered with their goal of dieting. Coricelli and colleagues argued that a similar conflict between enjoying food transformation and dieting could be what increased the reaction times of the restrained eaters in their study [see (11) for the background theory about such a conflict].

Restrained eating is considered to be a core symptom of anorexia nervosa (12). Interestingly, an interaction between an individual’s characteristics and food categorization in subjects suffering from anorexia nervosa has been documented by Urdapilleta et al. (3) in a social psychology study. The authors explicitly asked eating disorder patients (restrictive anorexic, binge/purge anorexic, and bulimic) and control subjects to categorize 27 food names. Results revealed that restrictive anorexic patients relied more on moral criteria (i.e., deontic terms such as obligation and permission “I can/cannot eat this”) to form food categories compared to other patients. This observation echoed religious asceticism that is historically deeply connected to what is sometimes called “holy anorexia”, illustrated by the case of Catherine of Siena or food deprivation that monks and clerics voluntarily endured in early Catholicism, anchored in ascetic practices defined at the end of Antiquity (13).

Morally-laden food perception and reasoning in anorexia nervosa has been highlighted in particular by Giordano (14), who put forward the idea that eating disorders are a particular expression of some moral beliefs. Especially anorexia nervosa could be driven by the pursuit of lightness and moral purity. Nowadays, words such as purity, decadence, heaven, and temptation are even recurrent in advertisements about food and in Western societies. The constant use of the lexicon of holy anorexia in advertisements has even been suspected to contribute to the maintenance of associations between eating certain foods and moral values, which might represent a risk factor of developing eating disorders (15). Interestingly, negative moral attributes such as “luscious”, “decadent”, and “temptation” in advertisements are generally associated with highly processed foods (14, 15). Furthermore, it has been suggested that similar mechanisms (e.g., disgust) might underpin the impurity judgments resulting from the transgression of moral laws, and the impurity judgments resulting from the transgression of regulation of eating or hygienic rules (16). The hypothesis that a same cognitive system anchored originally in distaste is now recruited by the moral domain would explain why some attributes might occur both in the food and the moral domain (e.g., lightness and purity). A similar theory that cultural domains such as morality invade older brain circuits such as disgust has been put forward by Dan Sperber [Sperber and Hirschfeld, (17)] and discussed in neuroimagery studies (18, 19).

This idea of an incursion of the moral judgment of food into the general population can be supported by the emergence of a specific eating attitude which has received a great deal of attention in recent decades: Orthorexia Nervosa, ON hereafter (20). This refers to an obsession about healthy eating that leads to emotional and psychosocial consequences such as anxiety and social isolation. Orthorexic traits are measured by self-declarative questionnaires, one of the most commonly used being the ORTO15 questionnaire (21). People suffering from ON exhibit a food restriction based on the healthiness and quality of food. Furthermore, they tend to exclude foods not considered sufficiently healthy or pure, two food attributes that seem to fall more into the category of pseudo-moral aspects than into the category of objective qualities of food (22).

The present studies aimed to determine whether certain food properties (especially those related to the energetic value of food) might trigger moral categories in subjects suffering from eating disorders and in the general population. More precisely, Study 1 aims to test whether patients suffering from anorexia nervosa (AN) would be more prone to lade food with moral properties than would the general population. Two specific research hypotheses have been tested in Study 1:

H1: Processed foods are implicitly associated with moral impurity whereas natural foods are associated with moral purity.

H2: Patients suffering from AN associate moral attributes with food more strongly than control subjects.

Study 2 further explored the relationship between food and moral attributes in the general population with and without orthorexia nervosa, by manipulating the objective calorie content (kcal/100 g) of the food instead of food processing as in Study 1. Two specific hypotheses were tested in Study 2:

H1’: High-calorie foods are implicitly associated with moral impurity whereas low-calorie foods are implicitly associated with moral purity.

H2’: Subjects exhibiting disordered eating behaviors associate moral attributes with food more strongly than control subjects.

## Study 1

### Method

#### Participants

A total of 75 participants completed the experiment. The patients with anorexia nervosa (AN group) were recruited by psychiatrists from three mental health units hosting patients suffering from eating disorders between March and August 2018. The inclusion criteria were (1) to be a woman aged from 18 and 35 years old, (2) to be diagnosed as suffering from anorexia nervosa (restricting or binge/purge types) according to the DSM-5 (23), (3) to not present any severe comorbidity (e.g., major depressive disorders), and (4) mastery of the French language. Moreover, participants with a BMI below 12 as well as those who were too heavily medicated (e.g., having a prescription of benzodiazepine that can alter reaction time), according to the psychiatrists, were not asked to participate. A total of 32 patients were included in the AN group, all with high education. All were diagnosed at least 1 year prior to testing, 2 were in remission, 17 were in relapse. The duration of the condition ranged from 1 to 18 years.

A first control group was formed from May to June 2018 with 32 students from the Paul Bocuse Institute, a school of management in hospitality and culinary arts, therefore students had background knowledge in nutrition and cooking. According to the literature, students in food-related studies, especially nutrition, have a higher prevalence (between 35 and 57%) of dysfunctional eating behaviors than the average of the general population (6.9%), particularly orthorexia nervosa (24, 25). Orthorexia nervosa appears to share a number of characteristics with anorexia nervosa, such as the presence of intrusive thoughts about food and a subordination of lifestyle and behavior to food imperatives (22). Considering these similarities and the fact that the present study focused on the relationship to food and on comparing healthy subjects with subjects suffering from AN, the orthorexic traits that were potentially present in the control group could bring a confounding variable to the study, and therefore needed to be assessed. The orthorexic traits of the students in the first control group were not tested. It was therefore decided to set up a second control group in the same population or in populations with a similar prevalence of orthorexia nervosa, such as medical students or students innutrition or agronomy, with an evaluation of orthorexic traits using the ORTO15 questionnaire. Participants included in the second healthy control group (HC group) were recruited through several email databases of French universities (AgroParisTech and Ecole Normale Supérieure Ulm) between May and July 2019. The inclusion criteria for the control group were (1) to be a woman from 18 to 35 years old and (2) to not present a potential eating disorder. This age group was targeted in order to have a sufficiently small age range to avoid a confounding factor of age on reaction times, and also to be able to compare the results of the HC group with those of a population suffering from anorexia nervosa (AN group), this mental illness affecting mainly adolescent and young adult populations. Of 43 respondents, 11 respondents presented eating disorder symptoms (i.e., with a score higher than the cut-off of 18 on the symptom index of the EDI-II short form) and were removed from the analyses. A total of 32 respondents were included in the HC group; they were students (65%) in agronomy, health, philosophy or psychology studies and employees (35%). A total of 64 participants were included in the analyses, 32 patients in the AN group and 32 in the HC group.

The experiment was approved by the local ethics committee (ID-RCB Number: 2015-A01194-45).

#### Measures

##### Participant Information

Data of patients with AN were collected through anonymous medical questionnaires filled out by the referring psychiatrist. This medical questionnaire comprises questions in order to document age, body mass index (BMI), type of anorexia nervosa, and other relevant anorexia nervosa-related information. Age and BMI of participants from the HC group were documented through anonymous questionnaires filled out by the participants themselves.

##### Eating Disorder Inventory II—Short Form

The short form of the Eating Disorder Inventory is a self-administrated questionnaire including 24 items that included 8 subscales (26). In this study, only symptom index score (mean score of the bulimia, body dissatisfaction, and drive for thinness subscales) was used. The respondent answered through a Likert scale ranging from 0 (Never) to 5 (Always). In the present study, Cronbach’s alpha (α) was 0.74. Only respondents in the HC group were asked to complete this questionnaire.

##### Food Questionnaire

The subject’s reaction time may be altered depending on the frequency of exposure to the food, which is itself related to its consumption. In order to avoid any recognition bias, the participants in the HC group filled out a questionnaire asking them to mention the foods they do not eat and the reasons why.

##### ORTO-15

ORTO-15 was used to assess orthorexic traits (21) among the HC group. The lower the scores, the higher the intensity of orthorexic behavior (21). All of the respondents in the HC group were asked to complete this questionnaire. The range of scores went from 31 to 43. In the present study, Cronbach’s alpha (α) was 0.56. During the development and validation procedure, ORTO-15 questionnaire reached satisfactory values for the cut-off point of 40 points (sensitivity = 100%, specificity = 73.6%, positive predicative value = 17.6%, and negative predicative value = 100%) (21). However, according to Dunn et al. (27) the frequency of ON as measured by ORTO-15 is too high. Cut-off point of 40 does not reflect the real prevalence of ON (28). Therefore, in some studies the cut-off point was lowered to 35 points (29, 30). In our study, 1 control subject had a score under 35, and 14 subjects had a score between 35 and 40. It is also important to mention that psychometric properties of the ORTO–15 scored as Donini et al. (21) suggested seemed to be poor (25, 31–33). Meule et al. (34) suggested that the poor psychometric properties of the ORTO–15 were largely due to the originally proposed scoring procedure. It consisted of having the items scored with the following response options: 1 = always, 2 = often, 3 = sometimes, 4 = never, except for six items: four of them were reversely coded (items #2, #5, #8, and #9) and two items (#1 and #13) had a rather unusual recoding procedure: 2 = always, 4 = often, 3 = sometimes, 1 = never. According to Meule and colleagues, who examined the psychometric properties of ORTO15 among 511 adults, principal component analysis revealed that only two items (#5 and #8) should be inverted, other items being scored as 1 = always, 2 = often, 3 = sometimes, 4 = never. After recoding, they found that internal reliability of the ORTO–15 items was acceptable (Cronbach’s α = 0.72) (34). Therefore, in the present study Meule and colleagues’ recommendations were followed.

##### Go/No-Go Association Task

A go/no-go association task (GNAT) described by Nosek and Banaji (35) was administrated to the participants through E-prime© software (Psychology Software Tools, Version 2.0 Professional). The GNAT assesses the strength of association between a target category and two poles of an attribute dimension (35). In this GNAT, the two target categories are natural food and processed food and the two poles of the attribute correspond to the notion of purity or impurity. Throughout the experiment, attributes referring to the notion of purity are called “pure words”, and those referring to the notion of impurity are called “impure words.”

Food stimuli were selected from the FoodPics database validated by Blechert et al. (36). Two sets of stimuli were created: one with 24 natural foods and the other with 24 processed foods, following Blechert and colleagues’ classification. Moreover, it has been shown that green might cue low energy density and that red is associated with a higher level of arousal compared to other colors (37). Thus, our two sets of stimuli (natural and processed) included the same proportion of green and red foods (12 green and 12 red food stimuli). To determine the extent to which these food variables are associated with the moral dimension of purity/impurity, we used a subset of attributes taken from a larger list of words constituted by Graham et al. (38). Graham and colleagues used the Linguistic Inquiry and Word Count program (LIWC; see Pennebaker et al. (39) to analyze liberal and conservative sermons. Then for each uses of these word, the consistency between the 2–3 sentences surrounding context of the word with the moral dimension (e.g., purity/impurity) was assessed by four independent raters who achieved a reliability of 0.79. Two sets of attributes were used in the present experiment, 12 attributes referring to moral purity and 12 attributes referring to impurity, to match the number of word attributes with the number of food stimuli and to have a balanced stimuli design. The stimuli are available in Supplementary Table 1.

**FIGURE 1:**
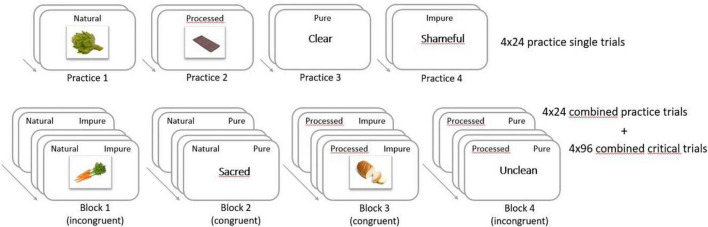
Design of the Go/No-Go Association Task to determine the strength of association between processed versus natural foods and morally “pure” versus “impure” attributes. In this study, the GNAT was administrated in French. For the purpose of this paper, this figure is an English translation of the task.

The GNAT included four practice single blocks, and four combined blocks (see Figure 1). For each block, participants had specific instructions. Depending on the instructions, participants were asked to press the space bar if they saw a stimulus in a specific target category, and not to press the bar if they saw any other stimulus.

The four practice single blocks consisted of two blocks with visual food stimuli, and two blocks with word stimuli. In the first practice block, participants had to press the bar if they saw a natural food on the screen, and not to press the bar if any other stimulus appeared on the screen (Practice 1), so the target category was natural food. In the second practice block, the target category was processed food (Practice 2), in the third practice block it was words associated with purity (Practice 3), and in the fourth practice block it was words associated with impurity (Practice 4).

The four combined blocks each had instruction aimed at two target categories. In Block 1, participants had to press the bar if they saw a natural food or an impure word on the screen, and not to press the bar if any other stimulus appeared on the screen, the target categories therefore being natural food and impure words (Block 1). Target categories for the second combined block were natural food and pure words (Block 2). For the third combined block, target categories were processed food and impure words (Block 3), and for the fourth combined block, processed food and pure words (Block 4). Among the four combined blocks, two were congruent blocks and represented the congruent condition, in which the association between the target categories was hypothesized to be stronger (Block 2 and Block 3). The two other blocks represented the incongruent condition, where the association between the target categories was hypothesized to be weaker (Blocks 1 and 4). For each block (practice or combined), distractor stimuli were the opposite of the target stimuli. For example, if the target stimuli were natural foods and pure words, then processed foods and impure words were both distractor stimuli.

Each practice block consisted of 24 stimuli with 12 stimuli from the target category and 12 distractor stimuli. Each combined block consisted of 120 trials with 120 stimuli, with first a familiarization phase and then a critical phase. The familiarization phase consisted of 24 stimuli with 6 training stimuli from each category of stimuli (i.e., natural food, processed food, pure words, and impure words). Then, following the same instructions, participants had to complete the critical phase consisting of 96 stimuli with 24 critical stimuli from each category of stimuli randomly presented to participants once each, with a ratio of 50% go stimuli and 50% no-go stimuli.

Each stimulus from the practice blocks and the combined blocks was visually presented for 1,000 and 850 ms (respectively) or until the participant decided to “go” and press the space bar. For the time window, a pre-test on 5 control subjects led us to choose a stimulus presentation duration of 850 ms, the performance obtained being relevant and consistent for this duration (error rate < 30%, success rate 84% on average) according to the literature (40, 41).

Prior to the task, participants were instructed to press the space bar of the keyboard as quickly as possible (GO) when the stimulus belonged to one of the two categories they were instructed to detect (e.g., Pure word or Natural food). If the stimulus did not belong to one of the target categories, then the participant had to inhibit the response (NO-GO). Emphasis was put on rapidity over accuracy. However, participants were also instructed to make as few mistakes as possible. Only for the practice single blocks, a green circle appeared on the screen when the participant had pressed the space bar when a target stimulus was shown (hit) or inhibited the response when a distractor was shown (correct rejection). A red cross appeared on the screen when the participant categorized a distractor as a target and pressed the space bar (false alarm) or missed a target stimulus by not pressing the space bar (miss). The green circle or the red cross were presented for 500 ms followed by a blank screen for 150 ms.

The reaction times (RT hereafter) in the practice single blocks and the RT in the familiarizing phase of each of the combined blocks were not recorded. Only RT in the critical phase were recorded and used in the statistical analyses.

#### Procedure

The experiment was conducted in a quiet testing room. The participants sat on a chair 70 cm from a liquid-crystal display (LCD) computer monitor with a resolution of 1,600 × 900 pixels (60 Hz refresh rate). After answering questions about which foods they did not eat and why, participants of both groups rated their state of satiety on a 7-point visual scale ranging from “not at all” to “extremely”. The GNAT instructions were verbally provided to participants by the experimenter and the GNAT was performed. To avoid the influence of task order highlighted by Nosek et al. (42), the order of the blocks was counterbalanced between participants. At the end of the experiment, the participants were asked to rate their level of familiarity of the words presented in the GNAT. The rating was made through a 5-point visual scale ranging from “Not known at all” to “Perfectly known.” The entire procedure took about 35 min.

#### Data Analysis

Analyses were conducted using Rstudio^®^ software (Version 3.6.0). Nosek and Banaji (35) and Greenwald et al. (43) recommend removing RT equal to or less than 300 ms as well as participants with more than 10% of trials faster than 300 ms. After examination, 19 trials met this criterion and were removed, and no participants were removed. Likewise, data were examined to verify that no participant exhibited an error rate greater than 40% on a given block or a 30% error rate overall. On the basis of these criteria, no participant was removed either. Reaction time and type of responses were recorded during the task. To analyze RT data, it was firstly screened for normality. The results of the Shapiro–Wilk [*W*(*142)* = 0.99, *p* = *0.387*] indicated normal distribution for RT means, results of Anderson-Darling for the residuals (*A* = 470.03, *p* < 2.2e-16) analysis of linear model with RT as dependent variable indicated a non-normal distribution of the residuals.

The mean and standard deviation of age, BMI, satiety score, and word familiarity scores were computed and compared between groups, and Spearman correlations were calculated to check for correlations between satiety scores and RT.

In order to test hypothesis H1, according to which food processing is implicitly associated with impurity whilst food naturalness is implicitly associated with purity, the RT were analyzed. As RT were normally distributed, Student tests were computed on RT, between the congruent associations and the incongruent associations in each group. With the same test, RT were analyzed between conditions (congruent versus incongruent) and groups, then between blocks to see whether an effect is driven by particular block(s). Power analysis was performed *post hoc* on each group with G*Power© software (44).

To measure the influence of group (AN or HC group) and condition (congruent or incongruent) factors on RT, a linear mixed model was conducted, because our data are repeated measures with the participant and the item as random factors. As the residuals are not normally distributed, a log transformation was made on RT. The models were constructed by iteratively adding predictive variables to the null model (M0, the intercept and no predictor), using the Akaike Information Criterion [AIC; (45)] as a basis for model selection. Group and condition were included in all models as fixed effects as well as possible interaction terms. Item and subject were included in all models as random effects. The R-squared (R^2^) was computed to determine the proportion of the variance explained by the model.

To test hypothesis H2, according to which the strength of the associations differ between AN and HC groups, D-measures were calculated as effect-size measures from the participants’ RT. Conceptually similar to Cohen’s d, the D-measure is the difference between the means of the RT in critical incongruent blocks and critical congruent blocks divided by the standard deviation of all the RT in these blocks (43). Since the D-measure does not seem to be improved by the deletion of responses faster than 400 ms in the Greenwald paper, all responses were kept.

### Results Study 1

#### Participants’ Characteristics

A total of 32 female participants with AN (Age: *M* = 24.40, *SD* = 4.7; BMI: *M* = 16.10, *SD* = 1.8) and 32 matched female control participants (Age *M* = 23.20, *SD* = 3.20; BMI: *M* = 20.8, *SD* = 1.9) were included in the analysis. The participants’ characteristics are presented in Table 1. Participants from the AN and HC groups did not differ in age, but differed in BMI. Results indicated also that state of satiety was significantly lower in the AN group. The Spearman correlation coefficient between state of satiety and RT (*Rhô* = −0.12, *p* = *0.403*) indicated that state of satiety was not significantly related to RT. The familiarity of the words did not differ between AN and HC groups.

**TABLE 1:** Study 1 participants’ characteristics by group and comparison of scores between groups.

Sample characteristics	AN group		HC group		*t*	*p*
	M	SD	M	SD		
Age	24.56	4.77	23.15	3.23	1.36	0.180
BMI	16.03	1.79	20.79	1.93	−10.21	<0.001
EDI-II-24	−	−	36.63	10.55	−	−
ORTO-15	−	−	39.38	4.04	−	−
Satiety score	2.09	1.58	3.31	1.79	−2.83	0.006
Word familiarity score	4.16	0.87	4.31	0.73	−0.77	0.405

*M, mean; SD, standard deviation; BMI, Body mass index; EDI-II, Eating Disorder Inventory—24 items; t, test statistic for the comparison test of each variable between the two groups; p, p value of each test.*

#### Level of Purity and Naturalness of Food

**FIGURE 2:**
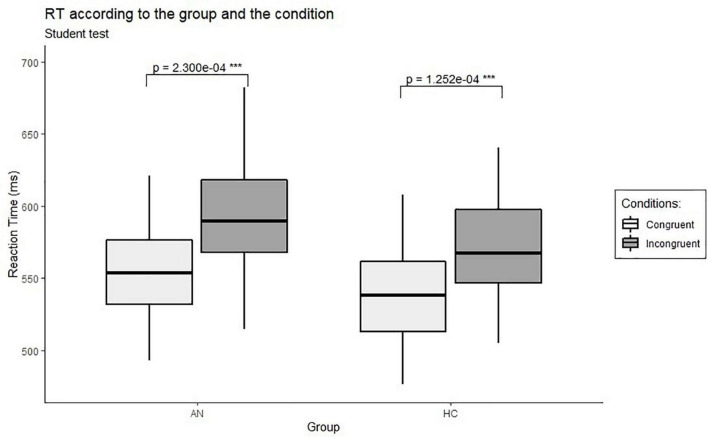
RT (ms) comparisons between conditions within-group. RT, reaction times; AN, AN group; HC, HC group. ***p < *0.001*, the difference between the two groups designated by the trait is significant.

In both groups, the means of RT in congruent conditions were significantly shorter than for incongruent conditions [AN group: *t*(63) = −4.12, *p* < *0.001*; HC group: *t*(62) = −4.30, *p* < *0.001*] (see Figure 2). This result was also found in each group (AN and HC group) with statistical powers of the association of 0.58 and 0.52 in each group, respectively. Then, the same analyses were conducted to compare RT between blocks for each group (see Supplementary Table 2). The means of the AN group’s RT were significantly shorter when natural foods were paired with words belonging to the pure moral category (Block 2) than when natural foods were paired with words belonging to the impure moral category (Block 1) [*t*(62) = −3.45, *p* = *0.012*, D-measure = 0.35]. The same result was found in the HC group: RT means were significantly shorter in Block 2 than RT means in Block 1 [*t*(61) = −4.26, *p* = *0.001*, D-measure = 0.38].

Concerning the attitude toward processed food, RT means were significantly shorter when processed foods were paired with words belonging to the impure moral category (Block 3) than when paired with words referring to the pure moral category (Block 4) [AN group: *t*(62) = −4.54, *p* < *0.001*, D-measure = 0.45; HC group: *t*(61) = −3.82, *p* = 0.003, D-measure = 0.35].

The mixed model conducted showed a significant effect of the condition [χ^2^(1,64) = 500.82, *p* < 2e-16] with the incongruent condition being significantly and positively different from the congruent condition [*beta* = 3.23, 95% CI (3.05, 4.08), *t*(10,481) = 16.18, *p* < *0.001*). The model showed neither an influence of the group on RT χ^2^(1,64) = 2.82, *p* = *0.093*], nor an influence of the interaction between the group and the condition [χ^2^(1,64) = 0.32, *p* = 0.572] on RT. Results are gathered in Supplementary Table 3. The model’s total explanatory power was: R^2^_*C*_ = 0.27.

D-measure (effect size) was also computed for each group according to the blocks and conditions. Results, presented in Supplementary Table 4, revealed that D-measures of each group were in the same target range, indicating a small effect size in all groups.

### Discussion, Study 1

The first hypothesis of this study (H1) was that food transformation is implicitly associated with impurity whereas food naturalness is implicitly associated with purity. Our results confirmed this hypothesis by revealing a facilitating effect on RT (shorter RT) in congruent compared to incongruent conditions. These results echoed Rozin and colleagues’ conclusions that consumers tend to exhibit a strong preference for natural foods over processed foods when they have the same chemical composition, the same taste, or when they are considered equally healthy (46). Indeed, according to Rozin and colleagues this preference could be grounded in beliefs that natural food would be purer and “morally superior” because it is “prior to human intervention” [(46), p.2]. However, these results seem to run counter to the findings of Coricelli et al. (47) that processed foods have been shown to trigger higher reward value and are more advantageous in terms of nutrients than unprocessed foods, so they have been favored as resource foods throughout evolution (47). Nevertheless, the study here explored the relation of food processing with morality, which is quite different from the nutritional aspects. Whereas processed foods are preferred in terms of taste and nutrients, morality speaking natural foods seemed to be more prone to be preferred as they are directly linked to nature and healthiness (46).

Furthermore, it is worth mentioning that the congruence effect does not result from the association between naturalness and purity only. This effect is also driven by the association between transformation and impurity. This result is consistent with the general belief that processed foods are more likely concealing unhealthy properties compared to natural counterparts. Such an unfavorable stance toward processed food could result from the principle of contagion, according to which the contact with an undesirable entity can render an object less desirable (48). Human intervention being considered to damage nature in modern Western societies (46), the contagion principle could lead one to associate processed food with negative moral attributes such as “decadent,” which are commonly used nowadays in advertisements (14, 15). Therefore, the association found in Study 1 between food transformation and morality corroborate the observations made by Rozin and colleagues. However, our findings revealed for the first time the existence of such an association at an implicit level. An association is automatic or implicit if it can occur even if participants do not have particular goals, a substantial amount of cognitive resources, a substantial amount of time or awareness (49, 50).

The second hypothesis of this study (H2) was that the strength of the implicit associations differs in patients suffering from anorexia nervosa and healthy control subjects. More precisely, and consistently with the literature on morally-laden food categories in patients with AN, we expected a stronger association in patients with AN than in healthy control subjects. As shown by the analysis of the D-measures and the generalized mixed model on the RT where no difference between groups was observed, the results did not confirm our second hypothesis.

#### Limitation and Perspectives, Study 1

One of the limitations could lie on the fact that the subjects included were all young women with high level of education. Therefore, no conclusion can be made for the general population regarding the results of the study. This choice was made because patients suffering from AN are described in the literature as mainly being adolescent or young women with high level of education (51, 52). Therefore, the population taken as a control group had to match these criteria in order for the two groups to be comparable.

Another limitation lied on the effect size of the mixed model: condition (congruent or incongruent) was considered to significantly influence reaction time, however, the effect size seemed to be relatively low: the incongruent condition being significantly and positively different from the congruent condition with an estimate of 3.23 [95% CI (3.05, 4.08)], compared to the intercept, which had an estimate of 1,445.32 [95% CI (1,433.29, 1,464.26)]. Therefore, these results should therefore be put into perspective.

Also, the initial ambition was to designed an implicit association task that was sensitive enough to capture individual characteristics of persons suffering from anorexia nervosa. Even if we confirmed the existence of an association between food transformation and morality, the strength of the association did not differ between control subjects and patients suffering from anorexia nervosa. One hypothesis why we might have failed to see such a difference lies in the food processing/naturalness variable, which might be a too subjective variable and therefore not the most appropriate here. We then decided to design a second task on the general population only to determine whether associations between objective energetic value and moral purity could be discriminant between ON and HC. This time we chose to test the second version on the general population before testing it on patients. Indeed, we wanted to confirm first that the task was properly calibrated and sensitive enough to capture disordered eating before using it to predict eating disorders relying on the assumption that if the task might detect ON it will detect a far more severe form of eating disorder.

Finally, the degree of processing is a subjective variable as it is highly dependent on the subject’s interpretation (53) and might therefore hide some subtleties about inter-individual differences in the studied association of moral attributes with food. Thus, a second study seemed necessary to disambiguate and extend the results found in Study 1.

## Study 2

According to Foroni et al. (37) who conducted a rating scale study in which participants were asked to rate the perceived calorie content and the arousal of food items, results reveal that the degree of processing is interpreted as an indicator of the energy density of food. The more processed a food is perceived to be, the more calories it is perceived to contain. In Study 2, we decided to conceptually replicate the association between energy density and moral categories by manipulating an objective food variable (calorie content per 100 g) as it is less open to interpretation by the subject and could help us to disambiguate the results generated by Study 1.

This replication was carried out using another technique measuring implicit associations: the Implicit Association Test (IAT). Indeed, as Nosek and Banaji (35) pointed out during the development of the GNAT, IAT and GNAT both measures the implicit attitudes toward concepts and attributes with the same variable (RT), and they tend to generate comparable results. The difference lies on the fact that the structure of the IAT constrains evaluations to be relative comparisons between two opposing categories, and therefore being a relative measure, whereas the GNAT allows for a separable assessment of categories, with framing evaluation of a target concept in a context of other concepts. As significant differences were found in Study 1 between congruent and incongruent blocks with the GNAT, we decided to replicate using an IAT in order to see if this technique would also show a significant difference between our categories in a relative comparison. Indeed, as the authors pointed out, “experimental reports that replicate implicit effects across techniques provide extra confidence that the effects are not due to a particular procedural aspect of any single tool” [(35), p.661].

As the present COVID-19 pandemic came across, the research had to be done online with the IATgen (54) and the Qualtrics (55) software.

### Method Study 2

#### Participants (Recruitment)

Participants were recruited through several French university mailing lists. The survey was circulated on June 1, 2021 and was available through June 30, 2021. Women and men from 18 to 35 years old were included. Indeed, as the prevalence figures show an equal proportion of men and women with orthorexia nervosa (27, 56, 57), men were first included in the recruitment. Of 180 respondents, 29 were excluded because of missing data and 8 were excluded due to aberrant response times. A total of 143 participants (116 women and 27 men) were included in the analysis. Participants were students (85%) in agronomy, health, or gastronomy studies; employees (5%); executives (9%); or inactive (1%). Four groups were formed: the “Orthorexic” group of participants (*N* = 21) with a high level of orthorexia-related symptoms (i.e., having an score on the ORTO-12-FR scale < 30), the “Pathologic” group of participants (*N* = 17) with a high level of eating disorder symptoms (i.e., having a score on the EDI-II-24 scale > 52), the “Ortho_Patho” group of participants (*N* = 43) with a high level of both orthorexia-related symptoms and eating disorder symptoms, and the “Control” group of participants (*N* = 62) not detected by either the ORTO-12-FR or the EDI-II-24 (score above 30 on the ORTO-12-FR and score below 52 on the EDI-II-24 scale).

#### Measures

##### Demographics Measures

The participants anonymously answered questions regarding their gender and age. They were asked to indicate their height and weight as well as their socio-professional category (58).

##### ORTO-12-FR

In this present study, ORTO-12-FR was used to assess orthorexic traits among the sample (59). ORTO-12-FR is a shorter French version of the ORTO15 developed by Donini et al. (21), with three items deleted after a confirmatory factor analysis (items 5, 6, and 8). All of the respondents were asked to complete this questionnaire. As in Study 1, Meule and colleagues’ recommendations (2020) (34) were followed for the scoring procedure. The range of scores went from 21 to 38. In the development of the ORTO-12-FR, no cut-off was established. However, Agopyan et al. (60) found that a cut-off of 30 could separate people exhibiting orthorexic traits (score below 30) and people without orthorexic traits (score above 30). As cut-off scores are not well established yet, we used both Agopyan and colleagues’ cut-off and ORTO-12-FR total score as a continuous variable. In the present study, Cronbach’s alpha (α) was 0.76.

##### Eating Disorder Inventory II - Short Form (EDI-II-24)

As in Study 1, participants completed this short form of the Eating Disorder Inventory including 24 items (26). In Study 2, Cronbach’s alpha (α) was 0.73. All of the respondents were asked to complete this questionnaire, and total scores ranged from 18 to 96. Respondents with a score higher than the cut-off of 52 (26), indicating the presence of an eating disorder or an unusual concern about body weight, were considered as pathologic.

##### Assessment of Their Satiety State

Participants were asked about their satiety level with a 7-point Likert scale ranging from “not hungry at all” to “very hungry”.

##### Implicit Association Task

**FIGURE 3:**
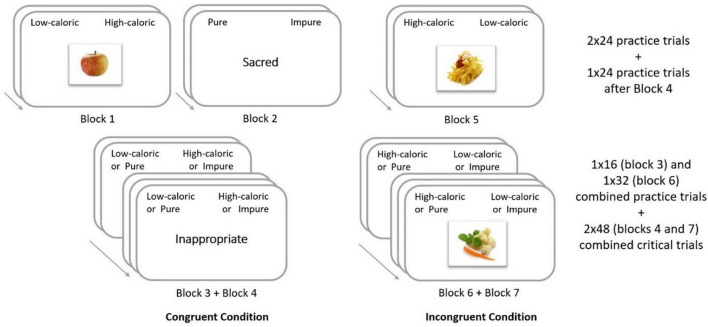
Design of the Implicit Association Test between low-caloric versus high-caloric foods and morally “pure” versus “impure” attributes. Interstimulus Interval (ISI): 1,650 ms in practice blocks with feedbacks, ISI: 1,150 ms in combined blocks. In this study, the IAT was administrated in French. For the purpose of this paper, this figure is an English translation of the task.

A slightly modified version of the IAT described by Greenwald et al. (61) was programmed with IATgen software (54). The IAT was then imported on Qualtrics© software. The IAT created was based on the original IAT described by Greenwald et al. (61) with further guidance from Greenwald (62). The first block of 24 trials consisted of practice on the calorie-content food classification task. The second block of 24 trials consisted of practice on the moral attribute classification task. The third and fourth blocks consisted of the first combined task (16 and 48 trials, respectively), including the classification of both foods and words related to morality. Half of the participants started with the same key for low-caloric food and impurity. For the other half of participants, the low-caloric food and words related to purity were initially associated with the same response key. The fifth block of 24 trials consisted of practice, this time for the low-caloric/high-caloric food classification task with reversed response key associations. The sixth block consisted of the second (reversed) combined task. As was suggested by Nosek et al. (42), the number of trials in this block was increased to 32 trials. The seventh and final block was made of 48 trials of the reversed combined task (see Figure 3 for a summary of the IAT blocks). It should be noted that blocks three and six served as practice for blocks four and seven, respectively. The participants completed 216 trials in total.

For the food stimuli, 24 food pictures were selected from the database FoodPics of Blechert et al. (36) with their energy density per 100 g and per stimulus (see Supplementary Table 5). Through this information, the selection of food stimuli was made to have two groups of 12 stimuli each, one representing low-caloric food and the other high-caloric food, and with the most contrasting averages and significant differences of kcal/100 g [*H*(1) = 252.00, *p* < *0.001*] and kcal/picture [*H*(1) = 256.00, *p* < *0.001*] between the low-calorie food and high-calorie food (see Table 2). Moreover, the selection was also made to ensure similar values within low-calorie and high-calorie food groups for both kcal per 100 g and kcal per stimulus.

**TABLE 2:** Study 2 means (M) and standard deviation (SD) Kcal per 100 g and Kcal per picture for each of the two groups of food stimuli constituted.

	Kcal per 100 g		Kcal per picture	
Food stimuli groups	*M*	*SD*	*M*	*SD*
Low-caloric	47.05	25.58	49.88	32.84
High-caloric	355.27	184.30	594.70	375.05

Regarding the word stimuli, the same 24 words selected from Graham et al. (38) in Study 1 were used: 12 words related to the notion of moral purity and 12 words related to the notion of impurity.

Participants were instructed to categorize as rapidly and accurately as possible the visual stimuli by pressing one of the two response keys (E or I) on the computer keyboard with their left and right index fingers. Emphasis was put primarily on rapidity over accuracy; however, the participants were instructed to also try and avoid errors as much as possible. Instructions about the mapping between the categories and the relevant response keys consisted of a schematic representation of the two response keys with the corresponding categories that was displayed on the screen. There was no time limit to learn the new categories–response mapping that remained in written form at the top-left and top-right corners of the screen as a reminder throughout each block of the experiment. In each trial, the participants started by looking at a fixation cross at the center of the screen for 1,000 ms. Then, a target stimulus was displayed. Feedback, consisting of a red cross, was provided after each incorrect target-response and remained on the screen for 500 ms. Each trial was separated by a blank screen corresponding to the inter-trial stimulus interval (ISI) of 1,000 ms. Participants’ RT and accuracy were recorded.

##### Post-test Categorization Task

Participants were asked to classify each stimulus as either low-caloric/high-caloric or pure/impure.

#### Procedure

After all participants gave their informed consent, participants were asked to answer gender and age questions. The IAT experiment was then performed by participants. To avoid the influence of task order (61), the key-response attribution of the qualifiers (“Low-caloric”/“High-caloric”; “Impure”/“Pure”) were counterbalanced across participants. Then, participants were asked to perform the post-test categorization task. Then, they completed the self-reported questionnaires (ORTO-12-FR and EDI-II-24) and some socio-demographic information. Finally, they indicated their satiety state. The entire procedure took about 15 min.

The procedure was in accordance with the Declaration of Helsinki and followed institutional ethics board guidelines for research on humans.

#### Statistical Analysis

##### Demographic Data Analysis

BMI was calculated from the height and weight reported by the participants. Pearson correlations were calculated between the BMI, the satiety level, the age, ORTO-12-FR score, and EDI-II-24 total scores.

##### IAT Analyses

All statistical analyses were performed using R. 3.6.0 studio software. The significance level was set to 5% (*p* < 0.05). According to Greenwald’s suggestions for improvement, RT under 300 ms or above 3,000 ms were also excluded. The normality of the RT distributions was checked with Q-Q plots and tested with the Shapiro test for each group in every block analyzed, which were the critical blocks (blocks 4 and 7). As the distributions did not follow the normality law, the Wilcoxon test was used to compare RT means in the two IAT conditions (congruent and incongruent) for each group. A Kruskal–Wallis test was also assessed to measure the differences between all groups.

To measure the IAT effect, D-measures were also calculated as effect-size measures from the participants’ RT. D-measures were computed as the difference between mean RT for blocks 3 and 6 (mean for block 6—mean for block 3) and blocks 4 and 7 (mean for block 7—mean for block 4), for which each resulting difference was divided by the pooled standard deviation of the two corresponding blocks.

A linear mixed model was also computed with RT (log-transformed) from the trials in which the participants responded correctly as the dependent variable, with the within-participants factors of Congruency (congruent associations: low-calorie food + word related to purity, high-calorie food + word related to impurity; incongruent associations: high-calorie food + word related to purity, low-calorie food + word related to impurity) and the Group (control, orthorexic, orthorexic and pathologic, pathologic) as the fixed effects. The participant number and the stimulus number were entered into the model as random effects. The models were constructed by iteratively adding predictive variables to the null model (M0, the intercept and no predictor), using the Akaike Information Criterion [AIC; (45)] as a basis for model selection. The R-squared (R^2^) was also computed to determine the proportion of the variance explained by the model.

As cut-off scores are not well established yet, ORTO-12-FR total score was also used as a continuous variable and additional generalized models were computed.

##### Post-test Analysis

The error rate of stimulus categorization was calculated per person, per group, and per stimulus type, and differences between groups and stimulus type were computed with Fisher’s exact test.

All of the statistical analyses above mentioned were also carried out without including men in the analyses (since we did not include them in Study 1). As no differences were found in the results, we decided to keep them in the sample analysis and results presented here.

### Results Study 2

#### Participants’ Characteristics

A total of 143 respondents were included in the analysis, aged from 18 to 35 years old (Age: *M* = 22.89, *SD* = 3.54; BMI: *M* = 21.92, *SD* = 3.15). Participants’ characteristics are gathered in Supplementary Table 6. Results indicate that state of satiety was not different from one group to another [*F*(3, 139) = 1.33, *p* = *0.269*]. The Pearson correlation coefficient between state of satiety and RT [*r*(141) = −0.03, *p* = *0.727*] ensures that state of satiety is not significantly related to RT.

No correlations were found between the demographic variables. As expected, a significant correlation was found between the ORTO-12-FR scores and the EDI-II-24 scores [*r*(141) = −0.43, *p* < *0.001*].

#### IAT Results

##### Results for H1

**FIGURE 4:**
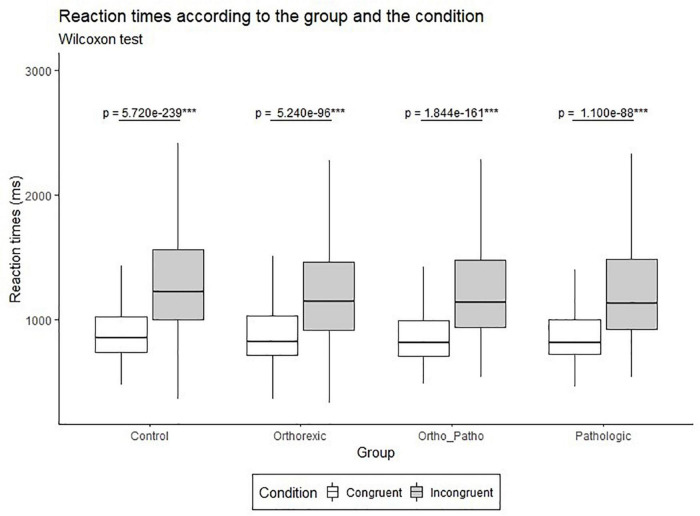
RT (ms) according to the group and the condition with Wilcoxon-test *p*-value results. ***Indicates significant differences between congruent and incongruent conditions.

Overall, mean RT was significantly different between the congruent and incongruent conditions (*U* = 2,115, *p* < *0.001*). This result was also found in each of the four groups (Control, Orthorexic, Ortho_Patho, Pathologic; see Figure 4) with statistical powers of the association of 0.99, 0.97, 0.99, and 0.93 in each group, respectively. Overall, the mean effect size was 0.86, with a standard deviation of 0.4. Detailed results are in Supplementary Table 7.

##### Results for H2

Overall, no significant difference was found between our four groups [*H*(3) = 1.68, *p* = *0.642*]. Mann–Whitney-tests between each pair of groups specify that no difference was found between groups. No significant difference between groups has been seen either regarding the effect size [*H*(*3)* = 3.05, *p* = *0.383]*.

The mixed model conducted showed a significant effect of the condition [χ^2^(1,143) = 3,564.95, *p* < 2e-16] with the incongruent condition being significantly and positively different from the congruent condition [*beta* = 14.17, 95% CI (13.88, 15.03), *t*(11,749) = 38.15, *p* < *0.001*]. The model showed neither an influence of the group [χ^2^(3,143) = 3.17, *p* = *0.366*] on RT, nor an influence of the interaction between the group and the condition [χ^2^(3,143) = 2.94, *p* = *0.401*] on RT. Results are gathered in Supplementary Table 8.

The model’s total explanatory power was: R^2^_*C*_ = 0.37.

This analysis conducted with the orthorexic score taken instead of the group variable did not show any significant influence [χ^2^(1,143) = 2.50, *p* = *0.114*].

#### Post-test Results

No significant difference between groups was shown [*F*(3,282) = 0.46, *p* = *0.708*] regarding the post-test results. Nevertheless, a significant difference regarding the type of stimuli was seen, with stronger error rates for food stimuli [Food stimuli: *M* = 0.94, *SD* = 1.4; Word stimuli: *M* = 0.17, *SD* = 0.4; *F*(1,284) = 41.2, *p* < *0.001*]. Overall, the mean error rates were really low, therefore stimuli were considered to be sufficiently correctly categorized for the IAT task.

### Discussion, Study 2

In this second study, we observed shorter RT in the congruent condition (block 4) than the incongruent condition (block 7) in all groups of participants. In addition, the calculation of the D-measure showed a large effect size in all groups. These findings support our hypothesis that high-calorie foods are implicitly associated with “impurity” whereas low-calorie foods are implicitly associated with “purity.” Moreover, this result extended our findings from Study 1 and suggest that both a subjective cue for energy content such as food transformation and an objective food variable such as calorie content per 100 g trigger moral attributes in healthy controls, subjects exhibiting orthorexia nervosa dispositions, and subjects exhibiting anorexia nervosa.

Stein and Nemeroff’s (1995) (63) analysis of a moralization of fat can shed light on the association found between high-calorie food and “impurity”. Indeed, in their study, the “fatty-food-eater” (people who eat “steak, hamburgers, French fries, doughnuts, and double-fudge ice cream sundaes” versus those who eat “fruit, especially oranges, salad, homemade wholewheat bread, chicken and potatoes”) were considered significantly less “moral” on a morality score composed of evaluations along dimensions such as considerate-inconsiderate, ethical-unethical, and kind-hearted-cruel on 8-point Likert-type scales.

Interestingly, Stein and Nemeroff obtained no evidence of a difference between restrained and unrestrained eaters in their moral inferences based on eating habits. In the same vein, hypothesis H2’ was not confirmed by our findings. The strength of the implicit associations was comparable between subjects exhibiting disordered eating behaviors and healthy control subjects: the analysis of the D-measures did not reveal any differences between the groups.

As a limitation, it should be noted that this experiment had to be done online due to the COVID-19 pandemic. Therefore, participants’ environments, which could have effects on reaction times, could not be controlled. Moreover, participants were young adults between 18 and 35 years old with high level of education, therefore, no conclusions regarding the general population can be drawn from the results.

## General Discussion

The present studies aimed to determine whether certain food properties might trigger such moral categories in the general population as well as in subjects suffering from eating disorders, without using declarative methods. Our findings revealed for the first time the existence of robust associations between food variables cueing energy value and moral attributes related to purity or impurity at an implicit level, in subjects suffering from eating disorders as well as in subjects exhibiting disordered eating behaviors and dispositions and control subjects. Furthermore, the studies reported here represent a first and successful attempt to capture the moral properties that various populations ascribed to food without relying on declarative data that might be liable to social desirability, declarative data being only used to described the population itself in these studies. In other words, they represent a first body of evidence that implicit methods might be fruitfully deployed to better understand moral categorization of foods in various populations.

In today’s Western societies, advertisers and marketers make extensive use of the vocabulary of morality when it comes to selling food products (15). Some foods that are usually highly processed and/or have a high calorie content have become “guilty pleasures” or “irresistible temptations.” At the same time, the development of nutrition education programs has contributed to the growth of the classification of foods into good and bad foods. Historically, moral adjectives were attributed to food when referring to people suffering from “holy anorexia,” also called “anorexia mirabilis” (i.e., people suffering from eating disorders using their religious beliefs to justify the way they eat and to protect themselves from judgments) (64, 65). Nowadays, the lexicon of morality seems to have pervasively influenced the manner in which the general population characterizes food. For instance, Brennan and colleagues (66) conducted recorded interviews with young adults about healthy eating. The interviews were so laden with moral terms that they decided to classify their participants into religious categories such as “Saint, Sinner, and Person in the Pew”. Study 1 and Study 2 revealed that these associations between moral categories and food variables are observable at an implicit level as well, in patients with anorexia nervosa, in subjects with orthorexia nervosa, and in healthy control subjects. Therefore, reasonable doubts about the idea that moralization of food would result only from social desirability or self-presentation concerns might be raised. Indeed, the measurement of robust implicit associations between moral attributes and food variables pave the way for further research on an evaluative system of categories about food that subjects cannot always control but that can still contribute to the expression of food behaviors and attitudes.

### Limits and Perspectives

An important limitation of our studies lies in the questionnaires used to categorize our participants into sub-groups. Firstly, the EDI questionnaire is made of different subscales that measure different dimensions of ED (drive for thinness, bulimia, body dissatisfaction, inefficacity, perfectionism, interpersonal distrust, interoceptive awareness, maturity fears). Here, only the EDI overall score was taken, as the sum of the scores for each dimension. Thus, anorexic as well as for instance bulimic symptoms have been taken into account. The inclusion of people with eating disorders other than AN may have reduced the effect size of the association, which may have been larger in only people with AN considering the previously discussed literature on AN. Nevertheless, no literature has been found about subjects with dietary disinhibition or binge eating concerning the association studied here. A promising perspective is thus to pursue the investigation of these associations between moral attributes and food variables in patients suffering from different eating disorders especially those characterized by a deficit of inhibition. Secondly, the ORTO15 was used to detect orthorexic traits. Even though it is the most widely used and translated measurement tool (67), several weaknesses have been raised such as its underlying structure, which was not assessed during its development (21), and its validity has been questioned with an overall accuracy of 0.70 (32). The corrected scoring procedure recommended by Meule et al. (34) showed internal consistencies of the ORTO15 and ORTO-12-EN of 0.56 and 0.76, respectively. These figures suggest that other tools may be more accurate in detecting orthorexia nervosa, but as new detection tools are under development, it seemed safer to use the most commonly used tool for these studies. Thirdly, it is important to note that these detections of orthorexia nervosa or eating disorder traits as well as the BMI of the participants were done with declarative data, which may present a social desirability bias. Indeed, as traits of eating disorders are not always well-regarded socially and even though the studies were anonymous, participants may have tended to respond in a way that they felt was more socially acceptable than their ‘real’ response, in order to project a favorable image of themselves, as described by Edwards (68). Thus, the formation of groups in Study 2 is to be put into perspective.

To conclude, these findings revealed that associations between food properties that cue for the energetic value of food triggered moral representations of purity/impurity in the general population, in the population suffering from disordered eating such as orthorexia nervosa, and in patients suffering from eating disorders such as anorexia nervosa. Further studies should try to explore whether such associations are also present at the opposite end of the disordered eating spectrum (i.e., loss of control) and whether such implicit associations have an impact on food behaviors on everyday food behaviors.

## Data Availability Statement

The datasets presented in this study can be found in online repositories. The names of the repository/repositories and accession number(s) can be found below: The databases and R code are available at https://osf.io/3eku8/?view_only=cf962fa223b2462ea8e2b9cc6b84d052 and upon request from the corresponding author.

## Ethics Statement

The studies involving human participants were reviewed and approved by CPP Ile-de-France; ID-RCB Number: 2015-A01194-45. The patients/participants provided their written informed consent to participate in this study.

## Author Contributions

JL, SI, VM, LT, and CL contributed to the conception and design of the study. SI, PD, VM, LT, and CL contributed to the acquisition of data. LT and CL organized the database and performed the statistical analysis. CL wrote the first draft of the manuscript. JL and MO wrote sections of the manuscript. All authors contributed to manuscript revision, read, and approved the submitted version.

## Conflict of Interest

The authors declare that the research was conducted in the absence of any commercial or financial relationships that could be construed as a potential conflict of interest.

## Publisher’s Note

All claims expressed in this article are solely those of the authors and do not necessarily represent those of their affiliated organizations, or those of the publisher, the editors and the reviewers. Any product that may be evaluated in this article, or claim that may be made by its manufacturer, is not guaranteed or endorsed by the publisher.
