# Supplementary material for: Sinful Foods: Measuring Implicit Associations Between Food Categories and Moral Attributes in Anorexic, Orthorexic, and Healthy Subjects
Source: Front Nutr. 2022 Jun 13;9:884003. doi: 10.3389/fnut.2022.884003 (PMC9234570; doi:10.3389/fnut.2022.884003)
Supplement: Supplementary file 1 [file Data_Sheet_1.docx]

# Supplementary Materials

## Supplementary Materials Table S1

**Table S1.** List of food stimuli in Study 1 from the database of Blechert and colleagues (2014).

| **Ref.**  **FoodPics** | **Name** | **Kcal per 100g** | **Kcal total** | **Red** | **Green** | **Degree of Transformation** |
| --- | --- | --- | --- | --- | --- | --- |
| 449 | garden radish | 15 | 4.5 | 0.40848 | 0.37694 | Natural |
| 460 | tomato | 17 | 13.6 | 0.75434 | 0.20539 | Natural |
| 257 | lettuce (lollo rosso) | 21 | 63 | 0.51129 | 0.25054 | Natural |
| 334 | carrots | 26 | 104 | 0.44464 | 0.42032 | Natural |
| 234 | strawberries | 31 | 9.3 | 0.59543 | 0.24827 | Natural |
| 401 | red chili | 40 | 4 | 0.65761 | 0.21229 | Natural |
| 192 | apple | 52 | 78 | 0.52422 | 0.31193 | Natural |
| 398 | cherries | 63 | 50.4 | 0.51320 | 0.21970 | Natural |
| 397 | grapes, red | 71 | 142 | 0.46819 | 0.24860 | Natural |
| 412 | potatoes | 71 | 142 | 0.47822 | 0.36872 | Natural |
| 539 | almonds | 598 | 478.4 | 0.53929 | 0.33371 | Natural |
| 450 | hazelnuts | 636 | 1157.5 | 0.53172 | 0.30414 | Natural |
| 264 | mushrooms (white) | 12 | 4.00 | 0.37022 | 0.33793 | Natural |
| 249 | cauliflower | 14 | 266 | 0.38006 | 0.40304 | Natural |
| 368 | chiccory | 15 | 105 | 0.38336 | 0.37402 | Natural |
| 455 | spinach | 17 | 13.6 | 0.33142 | 0.48762 | Natural |
| 360 | artichoke | 22 | 55 | 0.44306 | 0.45971 | Natural |
| 266 | green onion (shallot) | 42 | 44.1 | 0.36552 | 0.39099 | Natural |
| 466 | green apple | 52 | 114.4 | 0.39647 | 0.46946 | Natural |
| 261 | soybean sprouts | 52 | 41.6 | 0.37507 | 0.34690 | Natural |
| 282 | banana | 64 | 128 | 0.42835 | 0.36503 | Natural |
| 270 | corn (on a cob) | 67 | 388.6 | 0.39555 | 0.39551 | Natural |
| 281 | grapes | 71 | 71 | 0.42432 | 0.45206 | Natural |
| 283 | avocado | 217 | 542.5 | 0.46381 | 0.40153 | Natural |
| 253 | pickles | 16 | 36 | 0.35905 | 0.47814 | Transformed |
| 424 | peas cooked | 84 | 109.2 | 0.31149 | 0.50497 | Transformed |
| 385 | tagliatelle | 170 | 425 | 0.38425 | 0.34370 | Transformed |
| 560 | fried egg | 195 | 117 | 0.42245 | 0.34632 | Transformed |
| 300 | loaf of bread | 253 | 1265 | 0.44973 | 0.33740 | Transformed |
| 146 | cheese cake | 255 | 255 | 0.43560 | 0.34599 | Transformed |
| 182 | bowl of rice | 349 | 349 | 0.35472 | 0.32387 | Transformed |
| 226 | crisp bread | 350 | 700 | 0.37964 | 0.34406 | Transformed |
| 244 | rice waffles | 380 | 253.5 | 0.38688 | 0.33531 | Transformed |
| 515 | Emmentaler cheese | 383 | 766 | 0.40726 | 0.37653 | Transformed |
| 14 | muffin | 404 | 359.6 | 0.48600 | 0.36589 | Transformed |
| 294 | popcorn | 424 | 21.2 | 0.39837 | 0.35271 | Transformed |
| 361 | carrots, cooked | 26 | 39 | 0.57997 | 0.30674 | Transformed |
| 353 | strawberry tartlet | 104 | 111.3 | 0.66040 | 0.21426 | Transformed |
| 540 | beefsteak, raw | 111 | 333 | 0.55984 | 0.21914 | Transformed |
| 306 | roast potatoes | 117 | 269.1 | 0.57530 | 0.30807 | Transformed |
| 562 | cutlet | 133 | 399 | 0.52524 | 0.31806 | Transformed |
| 329 | salmon | 193 | 96.5 | 0.62898 | 0.26315 | Transformed |
| 549 | bagel | 233 | 186.4 | 0.52594 | 0.33257 | Transformed |
| 187 | croissant | 333 | 233.1 | 0.55322 | 0.28491 | Transformed |
| 348 | rusk | 365 | 36.5 | 0.52290 | 0.35202 | Transformed |
| 193 | crisp bread | 372 | 96.7 | 0.46632 | 0.35054 | Transformed |
| 152 | peanut puffs | 500 | 100 | 0.50156 | 0.34384 | Transformed |
| 286 | bar of chocolate | 530 | 530 | 0.51583 | 0.31211 | Transformed |

## Supplementary Materials Table S1’

**Table S1’.** List of word stimuli from the word bank of Graham and colleagues (2009).

| **Pure (12 words)** | | **Impure (12 words)** | |
| --- | --- | --- | --- |
| **English** | **French** | **English** | **French** |
| Clean | Propre | Lax | Laxiste |
| Innocent | Innocent | Sick | Malade |
| Pristine | Intact | Defile | Souillé |
| Wholesome | Sain | Gross | Dégoutant |
| Abstemious | Sobre | Wanton | Dévergondé |
| Saint | Saint | Contagious | Contagieux |
| Virgin | Vierge | Sinful | Honteux |
| Sterile | Stérile | Sins | Fautes |
| Refined | Epuré | Repulse | Répugné |
| Pure | Pur | Debase | Dégradé |
| Limpid | Limpide | Sinful | Coupable |
| Virtuous | Vertueux | Indecent | Indécent |

## Supplementary Materials Table S2

**Table S2.** Means and standard deviation of RT (ms) in each block in each group and comparisons between conditions within-group.

| **Group** | **Block1**  Natural food + Impure word | | **Block2**  Natural food + Pure word | | ***t*** | ***p*** | | ***D-measure*** | |
| --- | --- | --- | --- | --- | --- | --- | --- | --- | --- |
|  | ***M*** | ***SD*** | ***M*** | ***SD*** |  |  | |  | |
| AN group | 582.7 | 101.6 | 544.3 | 93.3 | -3.45 | | 0.012* | | 0.35 |
| HC group | 567.7 | 101.7 | 525.9 | 99.9 | -4.26 | | 0.001* | | 0.38 |
|  |  |  |  |  |  | |  | |  |
|  | **Block3**  Processed food + Impure word | | **Block4**  Processed food + Pure word | |  | |  | |  |
|  | ***M*** | ***SD*** | ***M*** | ***SD*** |  | |  | |  |
| AN group | 559.0 | 85.1 | 606.0 | 99.7 | -4.54 | | 0.000* | | 0.45 |
| HC group | 555.6 | 86.8 | 589.2 | 104.5 | -3.82 | | 0.004* | | 0.35 |

*Note.* AN, AN group; HC, HC group; t, test statistic; p, p-value of the Student test computed here between the two blocks and adjusted by the Bonferroni correction, D-measure, coefficient of the effect size. As guideline, a D-measure between 0.50 and 0.80 indicated ‘moderate’ effect; a strong effect is above 0.80 (Cohen, 1988; Nosek & Banaji, 2001).

** p-value < 0.05

##

## Supplementary Materials Table S3

**Table S3.** Summary of the generalized mixed model conducted on RT with the group and the condition (congruent/incongruent) as fixed effect, and with the subject and the stimulus as random effects in Study 1.

| **Effect** | **Parameter name** | ***Estimate*** | **95% CI** | | ***SD*** | ***p*** |
| --- | --- | --- | --- | --- | --- | --- |
|  |  |  | ***LL*** | ***UL*** |  |  |
|  |  |  |  |  |  |  |
| fixed | (Intercept) | 1445.32 | 1433.29 | 1464.26 | 0.59 | <0.001 |
| fixed | GroupHC | -1.10 | -2.96 | 0.00 | 0.72 | 0.123 |
| fixed | ConditionIncongruent | 3.23 | 3.05 | 4.08 | 0.20 | <0.001 |
| fixed | Interaction GroupHC: ConditionIncongruent | -0.16 | -1.00 | 0.00 | 0.28 | 0.572 |
| random | Stimulus | - | - | - | 2.91 | - |
| random | Subject | - | - | - | 2.80 | - |
| random | Residual | - | - | - | 7.33 | - |

Note. Number of observations: 10488; Number of subjects: 64. CI = confidence interval; LL = lower limit; UL = upper limit.

## Supplementary Materials Table S4

**Table S4.** D-measure (effect size) for each group according to the blocks and conditions.

| Group | D-measure | | |
| --- | --- | --- | --- |
|  | Blocks |  | Condition |
| AN group |  |  | 0.39 |
| NatImpur – NatPur | 0.35 |  |  |
| TransPur – TransImpur | 0.45 |  |  |
| HC group |  |  | 0.36 |
| NatImpur – NatPur | 0.38 |  |  |
| TransPur – TransImpur | 0.35 |  |  |

*Note.* AN, AN group; HC, HC group; NatImpur, Block 1; NatPur, Block 2; TransImpur, Block 3; TransPur, Block 4. According to Cohen’s d scale, a d of 0.2 corresponds to a small effect size, a d of 0.5 corresponds to a medium effect size, and a d of 0.8 corresponds to a large effect size.

## Supplementary Materials Table S5

**Table S5.** List of food stimuli in Study 2 from the database of Blechert and colleagues (2014).

| **Ref. FoodPics** | **Name** | **Kcal per 100g** | **Kcal total** | **Group Calorie content*** | **Degree of Transformation** |
| --- | --- | --- | --- | --- | --- |
| 274 | spinach | 17 | 17 | Low-caloric | Natural |
| 402 | pear | 52 | 78 | Low-caloric | Natural |
| 263 | mushrooms | 12 | 14.16 | Low-caloric | Natural |
| 280 | cherries | 63 | 37.8 | Low-caloric | Natural |
| 194 | kiwi | 53 | 26.5 | Low-caloric | Natural |
| 281 | grapes | 71 | 71 | Low-caloric | Natural |
| 369 | sushi roll | 100 | 40 | Low-caloric | Transformed |
| 558 | asparagus cooked | 20.5 | 114.8 | Low-caloric | Transformed |
| 567 | tomato and mozzarella | 86.7 | 104.04 | Low-caloric | Transformed |
| 362 | beans cooked | 25 | 30 | Low-caloric | Transformed |
| 249 | cauliflower | 14 | 266 | Low-caloric | Transformed |
| 196 | salad plate | 25 | 37.5 | Low-caloric | Transformed |
| 346 | potatoes | 71 | 426 | High-caloric | Natural |
| 459 | corn / maize | 211.2 | 528 | High-caloric | Natural |
| 283 | avocado | 217 | 542.5 | High-caloric | Natural |
| 457 | pine nuts | 559 | 335.4 | High-caloric | Natural |
| 539 | almonds | 598 | 478.4 | High-caloric | Natural |
| 282 | banana | 64 | 128 | High-caloric | Natural |
| 244 | rice waffles | 380 | 253.46 | High-caloric | Transformed |
| 181 | bowl of muesli | 343 | 857.5 | High-caloric | Transformed |
| 112 | opened bar of chocolate | 555 | 555 | High-caloric | Transformed |
| 366 | bagels | 276.1 | 496.98 | High-caloric | Transformed |
| 26 | chips | 539 | 183.26 | High-caloric | Transformed |
| 867 | pasta | 350 | 350 | High-caloric | Transformed |

*Note.* * group assigned by the authors.

## Supplementary Materials Table S6

**Table S6.** Study 2 Participants’ characteristics (number by gender, age, and BMI by groups, ORTO-12-FR, EDI-II-24, and Satiety scores with mean and standard deviation).

| Baseline characteristics | Control | | | | | Orthorexic | | | | Orthorexic &  Pathologic | | | | Pathologic | | | | |
| --- | --- | --- | --- | --- | --- | --- | --- | --- | --- | --- | --- | --- | --- | --- | --- | --- | --- | --- |
|  | n | | | | % | n | | | % |  | n | | % | n | | | | % |
| Number | 62 | | | |  | 21 | | |  |  | 43 | |  |  | 17 | | |  |
| Gender |  | |  | |  |  | |  |  |  |  |  |  |  |  | |  |  |
| Female | 45 | | | | 73 | 14 | | | 67 |  | 41 | | 95 |  | 16 | | | 94 |
| Male | 17 | | | | 27 | 7 | | | 33 |  | 2 | | 5 |  | 1 | | | 6 |
|  | *M* | | | | *SD* | *M* | | | *SD* |  | *M* | | *SD* |  | | *M* | | *SD* |
| Age ^1^ |  | 23.19 | | 3.80 | |  | 22.30 | | 2.31 |  | 22.37 | | 3.20 |  | | 23.82 | | 4.53 |
| BMI ^2^ |  | 21.66 | | 2.59 | |  | 21.02 | | 3.03 |  | 22.14 | | 3.08 |  | | 23.44 | | 4.71 |
| ORTO-12-FR ^3^ |  | 34.73 | | 2.89 | |  | 27.38 | | 2.29 |  | 25.77 | | 3.58 |  | | 33.41 | | 1.94 |
| EDI-II-24 ^4^ |  | 38.18 | | 8.98 | |  | 43.38 | | 9.07 |  | 65.95 | | 9.56 |  | | 57.65 | | 5.33 |
| Satiety score ^1^ |  | 2.16 | | 1.20 | |  | 2.62 | | 1.29 |  | 2.44 | | 1.44 |  | | 2.88 | | 1.50 |

*Notes.* BMI, Body mass index; EDI-II-24, Eating Disorder Inventory – 24 items

^1^ For Age and Satiety score, no significant difference between groups.

^2^ For BMI, only one significant difference between the Control group and the Pathologic group (*T* = -2.33, *p* = 0.028).

^3^ For ORTO-12-FR, the average score of each group is significantly different from that of the other groups.

^4^ For EDI-II-24, the average score of each group is significantly different from that of the other groups

except for the control and the orthorexic, where the difference is not significant between them (*T* = -1.29, *p* = 0.204).

## Supplementary Materials Table S7

**Table S7.** RT (ms) according to the group and the condition (means and standard errors), and Student-test results between RT of each condition in each group.

| Group | Conditions | RT (ms) | | Comparison between conditions | | |
| --- | --- | --- | --- | --- | --- | --- |
|  |  | *M* | *SD* | *t* | *p* | *D-measure* |
| Control |  |  |  |  |  |  |
|  | Congruent | 959.74 | 266.95 | -8.72 | < 0.001 | 0.84 |
|  | Incongruent | 1305.69 | 458.46 |  |  |  |
| Orthorexic |  |  |  |  |  |  |
|  | Congruent | 926.12 | 349.43 | -6.66 | < 0.001 | 0.85 |
|  | Incongruent | 1276.88 | 448.57 |  |  |  |
| Ortho_Patho |  |  |  |  |  |  |
|  | Congruent | 908.63 | 325.92 | -8.87 | < 0.001 | 0.92 |
|  | Incongruent | 1249.29 | 460.86 |  |  |  |
| Pathologic |  |  |  |  |  |  |
|  | Congruent | 933.36 | 335.52 | -5.44 | < 0.001 | 0.85 |
|  | Incongruent | 1316.09 | 491.83 |  |  |  |

*Note.* M, means; SD, standard error; t, test statistic; p, p-value of the Student test computed here between the two conditions and adjusted by the Bonferroni correction; D-measure, coefficient of the effect size. As guideline, a D-measure between 0.2 and 0.50 indicated a ‘small’ effect; a D-measure between 0.50 and 0.80 indicated a ‘moderate’ effect; a strong effect is above 0.80 (Project Implicit, 2017).

## Supplementary Materials Table S8

**Table S8.** Summary of the mixed model conducted on log-transformed RT with the group and the condition (congruent/incongruent) as fixed effect, and with the subject and the stimulus as random effects in Study 2.

| **Effect** | **Parameter name** | ***Estimate*** | **% CI** | | ***SD*** | ***p*** |
| --- | --- | --- | --- | --- | --- | --- |
|  |  |  | *LL* | *UL* |  |  |
| fixed | (Intercept) | 1832.21 | 1791.58 | 1868.78 | 0.86 | <0.001 |
| fixed | ConditionIncongruent | 14.17 | 13.88 | 15.03 | 0.35 | <0.001 |
| fixed | GroupOrtho_Patho | -1.58 | -4.88 | 1.01 | 1.58 | 0.310 |
| fixed | GroupOrthorexic | -2.17 | -4.88 | 0.00 | 1.24 | 0.075 |
| fixed | GroupPathologic | -1.00 | -3.92 | 2.02 | 1.72 | 0.555 |
| fixed | ConditionIncongruent: GroupOrthorexic | 0.75 | -1.00 | 2.02 | 0.69 | 0.276 |
| fixed | ConditionIncongruent: GroupOrtho_Patho | 0.22 | -1.00 | 1.01 | 0.54 | 0.685 |
| fixed | ConditionIncongruent: GroupPathologic | 1.13 | 0.00 | 3.05 | 0.74 | 0.129 |
| random | Id | - | - | - | 6.10 | - |
| random | Stimulus | - | - | - | 2.26 | - |
| random | Residual | - | - | - | 13.06 | - |

Note. Number of observations: 11760; Number of subjects: 143. CI = confidence interval; LL = lower limit; UL = upper limit.
